# Moss spores: overlooked airborne bioparticles in an urban environment

**DOI:** 10.1007/s11356-024-35028-5

**Published:** 2024-09-21

**Authors:** Jana Ščevková, Mária Tropeková, Jozef Dušička, Natália Štefániková, Matúš Žilka, Eva Zahradníková, Jozef Kováč, Katarína Mišíková

**Affiliations:** 1 Faculty of Natural Sciences, Department of Botany, Comenius University, Révová 39, 811 02 Bratislava, Slovakia; 2 Faculty of Mathematics, Physics and Informatics, Department of Applied Mathematics and Statistics, Comenius University, Mlynská dolina, 842 48 Bratislava, Slovakia

**Keywords:** Aerospores, Bryophyta, Aerobiology, Urban space, Slovakia

## Abstract

Moss spores are present in aerobiological samples, but their low representation, lack of known allergenic properties, and difficult identification have led to their being overlooked by aerobiologists so far. The data about their presence in the atmosphere and the factors that influence them are, however, important from the biodiversity conservation point of view, since they give us information about their ability to spread to new habitats. In this pilot study, we analysed their presence in the atmosphere of Bratislava city, Slovakia (2018–2023), using Burkard volumetric sampler, and determined the most significant factors influencing its temporal distribution. The size category of 13–18 µm was the most represented in the samples. Environmental factors influenced the daily spore concentrations and the characteristics of the whole spore season. The start of the Main Spore Season (MSS) depended mainly on the temperatures in November–January, initiating earlier growth of sporophytes in the following year, while the intensity of the MSS was influenced by high humidity in April, stimulating the formation of spores in sporangia. The daily concentration of airborne moss spores was mostly influenced by the actual temperature and wind speed, promoting the release and dispersal of spores, and precipitation lowering their levels due to the “wash-out” effect, although no intradiurnal pattern was observed. More data from other locations is needed to determine the role of atmospheric spore transport for the conservation of moss species facing anthropogenic climatic change.

**Supplementary Information:**

The online version contains supplementary material available at 10.1007/s11356-024-35028-5.

## Introduction

Mosses (division Bryophyta) are non-vascular spore-bearing plants, consisting of some 12,000 species worldwide (Goffinet & Buck 2004). These organisms with a long evolutionary history thrive in various climates and habitats demonstrating remarkable adaptability, classifying them as cosmopolitan organisms. In general, they prefer shady or humid habitats, but many species are adapted to sunny, seasonally dry areas (Erkara et al. 2018). Their species richness decreases with latitude, with the highest biodiversity found in tropical regions (Qian et al. 2024).

Mosses reproduce asexually by production of vegetative propagules or fragmentation of gametophytes and sexually by meiospores which are passively dispersed in the environment predominantly by air currents (Hutsemekers et al. 2008). Most moss spores are spherical and generally small, 5–20 μm in diameter (Frahm 2009), which should give them good aerodynamic properties and buoyancy observed in bioparticles of similar size (some pollen grains and fungal spores), making them an integral part of the atmospheric microbiome (Fröhlich-Nowoisky et al. 2016). Like in other aerospores, the internal structures of moss spores are protected by a resistant sporoderm containing sporopollenin, sheltering them from environmental stresses (Boros et al. 1993) and enabling their survival in harsh atmospheric conditions (strong UV radiation, temperatures much below the freezing point, drought, chemical air pollutants). Additionally, the moss spore envelope may include, besides exine and intine, an outer perine layer with perine elements. This layer can possibly be filled with air and improve the ability to float (Polevova et al. 2019). Nevertheless, our understanding of the aerobiological pathway of these highly efficient wind-dispersed organisms (Patiño & Vanderpoorten 2019) remains limited, as moss spores receive minimal attention from aerobiologists in comparison to pollen grains and fungal spores (Marshall & Convey 1997; Miller & McDaniel 2004; Sundberg 2013; Essien 2019). Unlike the above-mentioned bioparticles, it is believed that they contain no allergenic molecules with the potential to trigger allergenic respiratory diseases in sensitive individuals (Martínez-Abaigar & Núñez-Olivera 2021). However, further research can reveal new findings in this area.

The concentration of airborne moss spores depends primarily on meiospore production of the parental organisms, the mechanism of their discharge from the capsule and meteorological conditions. In general, moisture is required for the fertilization process and formation of sporophytes. Evidence of high spore production for species with small spores (7–20 μm) is evident; for instance, a single sporangium in *Sphagnum* may release up to 243,000 spores (Sundberg & Rydin 1998), while in *Dawsonia*, the count can soar to 5 million spores (Frahm 2001). Considering the discharge of spores from sporangium, certain moss species exhibit active release mechanisms, where meiospores are forcefully ejected from capsules (Sundberg 2010). In contrast, the majority of them have passive spore release through wind-induced vibrations of the sporangium (Johansson et al. 2014). Based on these factors, the highest airborne spore concentration should be reached in warm, dry weather with dynamic air circulation, when dry sporangia open and the released spores can easily overcome the boundary layer and enter the free atmosphere (Wiklund & Rydin 2004). Regardless of the mechanism of spore release, most spores do not reach a distance from the source plant greater than a few hundred meters (Snäll et al. 2005; Löbel et al. 2009), especially in closed forest conditions. However, their location in open environments with unobstructed airflow or at higher elevations (where they have greater chances of becoming airborne), enables them to be involved even in long-distance atmospheric transport over hundreds of kilometres (Marshall & Convey 1997; Miller & McDaniel 2004; Sundberg 2013; Barbé et al. 2016; Mota de Oliveira et al. 2022).

Despite the inaccuracy of the widely accepted statement that mosses only absorb water and nutrients together with the pollutants from the atmosphere (Varela et al. 2023), air pollution can influence their growth and spore production. The increased levels of CO_2_ improve biomass growth and productivity in mosses (Cerff & Posten 2012) and could lead to increased spore production like in vascular plants (Ziska 2021). Other atmospheric pollutants, like O_3_, NO_2_, CO, and SO_2_ can act as plant stressors (Oksanen & Kontunen-Soppela 2021).

Despite the decades-long permanent aerobiological monitoring, attention is primarily focused on pollen grains and fungal spores and information about other bioparticles in the atmosphere, including moss spores, and their spatio-temporal variability is lacking. Therefore, this study aims to conduct a pilot spatio-temporal examination of airborne moss spore occurrence within the inland temperate climate with regards to their size. Additionally, the effects of meteorological parameters and air pollutants on their levels in the air were assessed.

## Materials and methods

### Study area

**Fig. 1:**
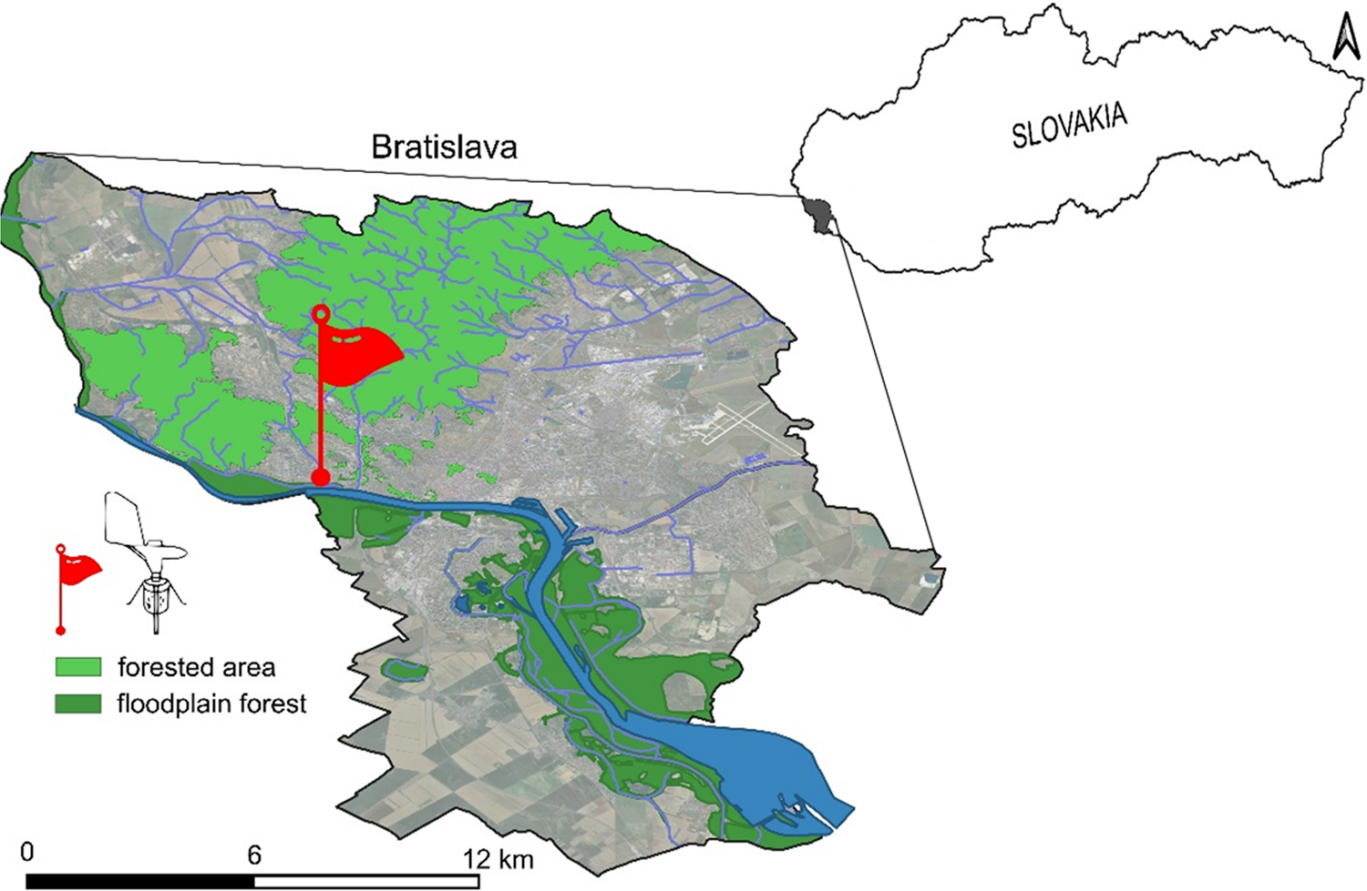
Location of spore sampler in Bratislava. Forested areas are mainly represented by Carpathian deciduous forests

Aerobiological samples were collected in the city of Bratislava (48°08′ N, 17°06′ E, 152 m a. s. l., 367.7 km^2^) located in southwestern Slovakia at the foothills of the Malé Karpaty Mountains (Fig. 1). The greenery in Bratislava covers 40% share of the total area of the city (Belčáková et al. 2022). Commercial forests, the remnants of natural forests and forest parks account for 91% of green spaces in the city. There are the Carpathian oak-hornbeam and beech tree forests (especially in the northern part of the city) and floodplain forests (especially in the southern part of the city). The remaining green spaces are parks, ornamental gardens, lawns and alleys.

The climate in the study area is temperate continental. The detailed descriptions of the weather conditions in Bratislava can be based on meteorological data from the period 2010–2023 obtained from the Bratislava–Mlynská dolina meteorological station (located 500 m apart from the aerobiological sampler). The mean annual air temperature was 11.4 °C with the highest mean monthly temperature recorded in July (22.0 °C) and the lowest in January (0.3 °C). The average precipitation is 602 mm/year with the highest rainfall in the May˗September period. Over the studied years (2018–2023), the highest mean annual temperature (12.2 °C) and precipitation (1170.9 mm) were recorded in 2023. The lowest temperature (10.8 °C) was recorded in 2021 and the lowest precipitation (830.4 mm) in 2022.

Despite the relatively dry and warm climate and low altitude, 245 bryophyte taxa have been recorded in the study area (Janovicová et al. 2003). The richness of species is influenced by the location of the city at the borders of four phytogeographical districts of Slovakia (Záhorská and Podunajská nížina Lowlands, Devínska Kobyla and Malé Karpaty Mountains), as well as the variability of geological bedrock and biotope diversity. Of the different biotope types, the highest species diversity was found in Carpathian deciduous forests (102 species), while only 56 species were found in lowland floodplain forests (Janovicová et al. 2003; Jančovičová et al. 2022). The most common species in the Carpathian forests are sporophyte-forming terrestrial mosses, including *Amblystegium serpens*, *Atrichum undulatum*, *Brachytheciastrum velutinum*, *Brachythecium rutabulum*, *Ceratodon purpureus*, *Dicranella heteromalla* and *Polytrichum formosum* and epiphytic *Hypnum cupressiforme*. The spectrum and quantity of mosses in the lowland floodplain forests are lower due to the high vegetation cover of vascular plants. Epiphytic species are more common here, including the sporophyte-forming *Hypnum cupressiforme*, *Leskea polycarpa* and genera *Orthotrichum* and *Lewinskya*. Mosses in the built-up spaces of the city grow mostly on anthropogenic surfaces, with the most common sporophyte forming genera *Barbula*, *Bryum* s. l.*, Didymodon*, *Schistidium* and species *Ceratodon purpureus*, *Funaria hygrometrica*, *Grimmia pulvinata* and *Tortula muralis*. The nomenclature of mosses follows Hodgetts et al. (2020).

### Aerobiological data

The concentration of airborne moss spores was measured from 1 March to 30 November (275 daily samples per year) for six consecutive years (2018‒2023), using a Hirst-type volumetric aerospore trap (Burkard Manufacturing Co Ltd.). The sampler was located on the rooftop (N 48.14973, E 17.07375) at 18 m a. g. l., thus avoiding the barrier effect of urban development on airflow. At this altitude, the air is sufficiently mixed and, in addition to aeroparticles of local origin, also contains those that are remotely transported (Lacey & West 2006).

The volumetric sampler sucks in 10 l of air per minute. The air inside hits an adhesive tape placed on a rotating drum, where aeroparticles are captured on a vaseline coating. The drum revolves with a speed of 2 mm/h and turns completely in 7 days, determining the changing interval of the exposed tape. The tape was then cut into segments, each representing a daily exposure, and microscopic slides were prepared by mounting them in glycerol gelatine and staining with fuchsin. Moss spores were identified and counted along twelve vertical transects per slide, each corresponding to two hours, using a light microscope with a magnification of 400 × (Galán et al. 2007). The average daily concentrations of moss spores per cubic meter of air (spores/m^3^) were calculated using a standard formula, considering the volume of sampled air and exposition time, presented by Lacey & West (2006). Bryophyte spores were distinguished from other aerobiological particles by their lack of staining with basic fuchsine, which differentiates them from pollen grains (Galán et al. 2007), and by comparing their morphological features to those described in the literature (Boros et al. 1993; Lacey & West 2006) and to spores from herbarium specimens available in the SLO herbarium. Fungal spores were also identified in the aerobiological samples using routine monitoring methods to improve the accuracy of bryophyte spore identification. Spores that could not be definitively identified were not counted. The most important morphological characteristic of moss spores, deciding their ability to spread into new areas and compensate for the habitat loss caused by anthropogenic climate change, is their size. Smaller size leads to a lower sedimentation velocity, letting them cover a greater distance. The moss spores found in the aerobiological samples were grouped by their size: (i) ≤ 12 µm in diameter, (ii) 13–18 µm and (iii) ˃ 18 µm, with the medium size group (ii) covering 90% of the sampled spores, and the remaining spores being either smaller (i) or bigger (iii).

The Main Spore Season (MSS) was determined as the period that includes 90% of the total annual spore concentration (Annual Spore Integral – ASIn). Therefore, the MSS signifies the interval during which the cumulative sum of mean daily spore concentrations ranges from 5 to 95% of the total annual spore concentration (Nilsson & Persson 1981) and the airborne spore concentration reached during this time is the Seasonal Spore Integral – SSIn.

### Environmental data and statistical analysis

The effects of the environmental variables on moss spore concentration during the MSS in Bratislava were analysed using Poisson regression with a logarithmic link function. The meteorological data (daily mean air temperature (°C), relative humidity (%) and wind speed (m/s), sunshine (h) and total daily precipitation (mm)) and air pollution data expressed in μg/m^3^ for O_3_ – ozone, CO – carbon monoxide, NO_2_ – nitrogen dioxide and SO_2_ – sulfur dioxide) utilised for the statistical analyses were sourced from the Slovak Hydrometeorology Institute (SHMÚ). To address variations across years, a factor variable “Year” (with levels 2018, 2019, 2020, 2021, 2022 and 2023) was also included in the model. Additionally, to correct for autocorrelation in the measurements, we also incorporated lagged observations of the response variable in the model as suggested in Liboschik et al. (2017), more precisely, we used model (1) from their paper, in our case of the form

$${\uplambda }_{\text{t}}=exp\left({\upbeta }_{0}+{\upbeta }_{1}\mathit{log}\left({y}_{t-1}+1\right)+{\upbeta }_{2}\mathit{log}\left({y}_{t-2}+1\right)+{\upeta }_{1}{x}_{t,1}+{\upeta }_{2}{x}_{t,2}+\dots +{\upeta }_{r}{x}_{t,r}\right)$$

where ${y}_{t}$ is the value of the response variable at time $t$, ${x}_{t,1},\dots ,{x}_{t,r}$ are the values of the explanatory variables at time $t$, ${\uplambda }_{t}$ is the parameter of the Poisson distribution at time $t$ and ${\upbeta }_{0},{\upbeta }_{1},{\upbeta }_{2},{\upeta }_{1},{\upeta }_{2},\dots ,{\upeta }_{r}$ are the estimated parameters of the model. Note that if ${\upbeta }_{1}={\upbeta }_{2}=0$ (i.e. if we omit the lagged observations), we get a standard Poisson regression.

The number of previous observations (in this case two) was chosen to be the smallest possible in such a way that the residuals of the final model did not exhibit a significant correlation. Estimates of the model parameters were obtained using R software using the *tsglm* function from the *tscount* package. Finally, we sequentially removed variables to optimise the Akaike Information Criterion (AIC) with a standard backward elimination technique. This means that we started with a model with all variables and removed the variable whose omission most improved the AIC. We repeated this elimination iteratively until we arrived at a model in which the removal of any variable would cause a worsening of the AIC. The variables Beta1 and Beta2 represent the effect of the value of the response variable shifted back by one and two days, respectively^1^. The impact of the year 2018 is included in the intercept and the variables of type 20**‒2018 reflect the difference between the impact of that year and the impact of 2018. The remaining variables directly represent the effect of the environmental variables on spore levels.

## Results

**Fig. 2:**
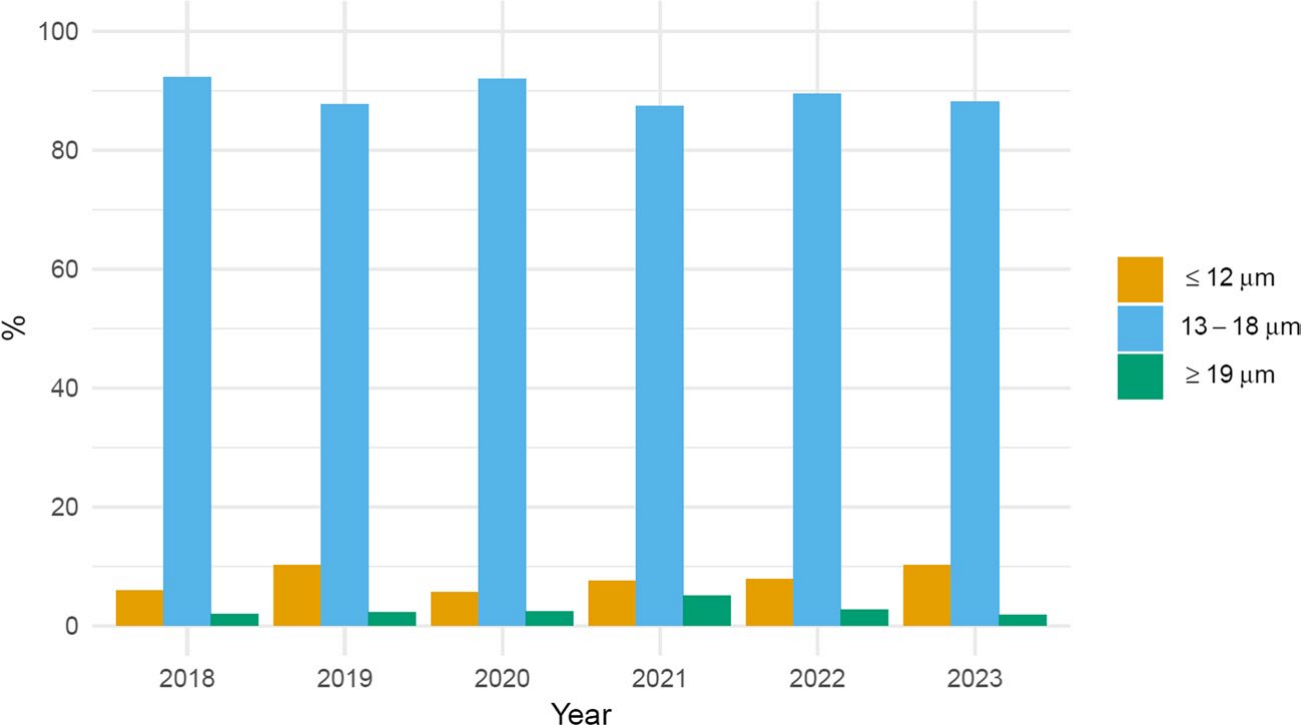
Percentage contribution of moss spores (based on their size) in the air of Bratislava in individual years

**Fig. 3:**
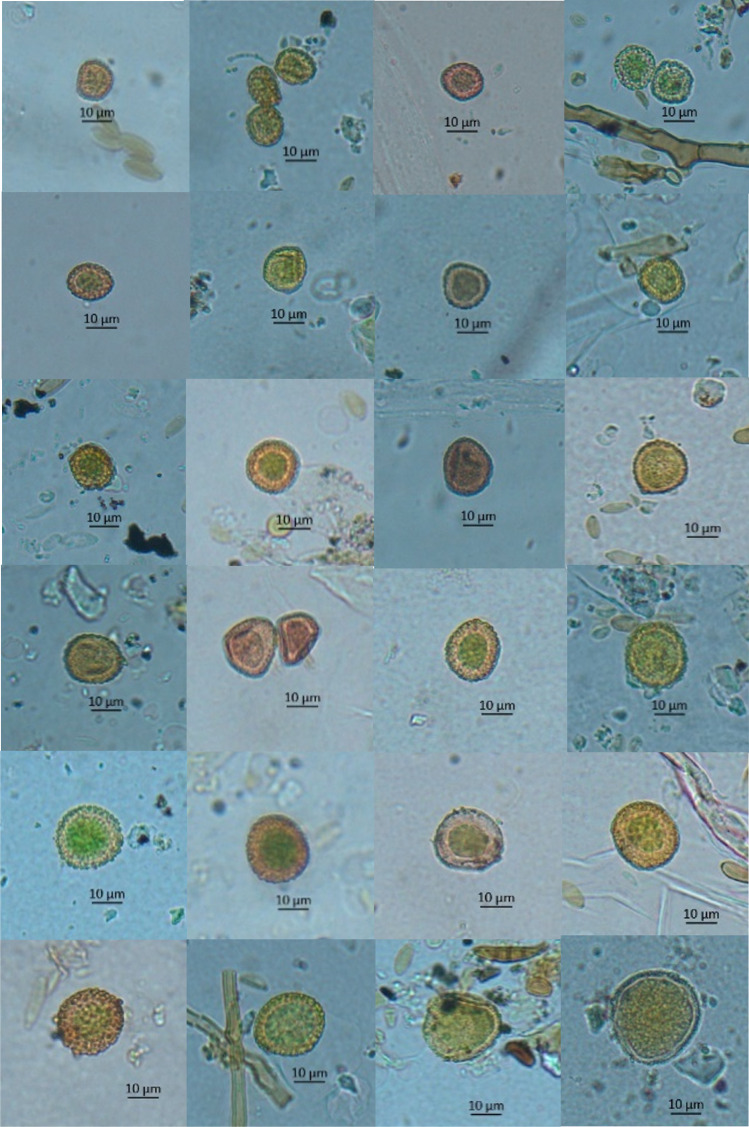
Images of moss spores with a diameter from 12 to 30 µm from a light microscope (magnification 400 ×) sampled in the air of Bratislava

**Fig. 4:**
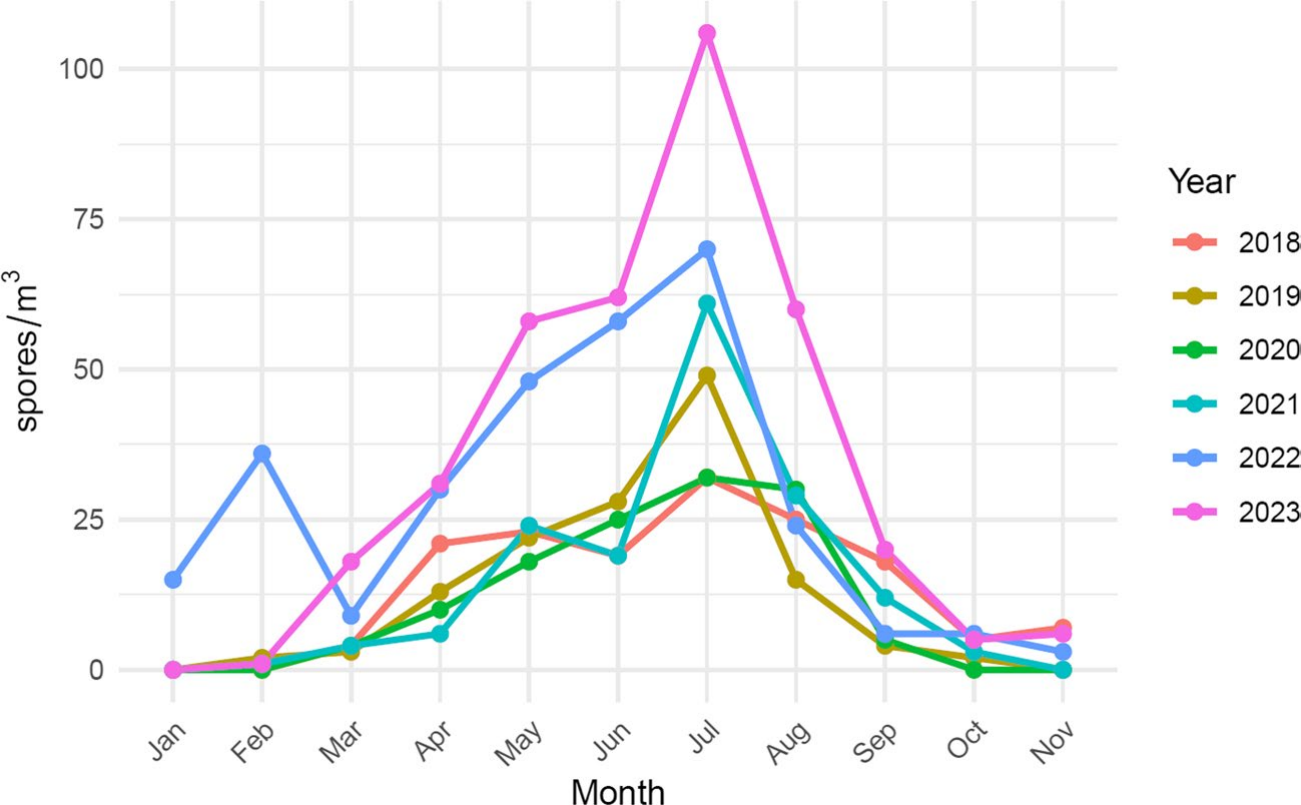
Monthly variation in airborne moss spore concentrations in Bratislava, 2018–2023

The average ASIn of mosses (2018–2023) in the study area was 211 spore*day/m^3^. The highest value, reaching 366 spore*day/m^3^, was observed in 2023, while the lowest, 124 spore*day/m^3^, occurred in 2020 (Table 1). The most abundant size category of spores in the samples was 13–18 µm in diameter (between 87.2% of the total spore concentration in 2023 and 92.2% in 2018), followed by spores ≤ 12 µm in diameter (between 5.7% in 2020 and 10.3% in 2019) (Table 1, Fig. 2). Spores greater than 18 µm were only sporadically present in the samples. The size categories are visible in Fig. 3. Based on the list of sporulating mosses identified in the study area, spore size and exine surface ornamentations, we hypothesise that the dominant spores belong to the species *Amblystegium serpens*, *Brachythecium rutabulum*, *Ceratodon purpureus*, *Funaria hygrometrica*, *Hypnum cupressiforme*, *Leskea polycarpa*, *Orthotrichum diaphanum* and *Schistidium apocarpum* (Table S1). The highest spore concentrations were recorded in July, from 32 spores/m^3^ in 2018 and 2020 to 106 spores/m^3^ in 2023 (Fig. 4).

**Table 1:** Total monthly/annual spore concentrations (spores/m^3^) of mosses (Bryophyta) in the air of Bratislava

Year	Spore size (µm)	Month									Total
		III.	IV.	V.	VI.	VII.	VIII.	IX.	X.	XI.	
2018	≤ 12				4	2	3				9
	13–18	4	21	21	15	29	22	18	5	7	142
	˃ 18			2		1					3
2019	≤ 12			2	6	5	1				14
	13–18	3	12	20	21	43	14	4	2		119
	˃ 18		1		1	1					3
2020	≤ 12			1	2	4					7
	13–18	4	10	16	23	26	30	5			114
	˃ 18			1		2					3
2021	≤ 12			2	1	8	1				12
	13–18	4	6	19	17	49	28	12	3		138
	˃ 18			3	1	4					8
2022	≤ 12		2	6	7	5	2				22
	13–18	9	28	40	47	63	22	6	6	3	224
	˃ 18			2	4	2					8
2023	≤ 12		2	6	5	14	5	1			33
	13–18	18	27	50	54	86	54	19	5	6	319
	˃ 18		2	2	3	6	1				14

The results of the aerobiological analysis show that moss spores are present in the air of the study area throughout the whole monitoring period. On average, their MSS starts at the beginning of April (Table 2). The earliest MSS start was in 2023 (31 March), 11 days earlier than the latest start in 2019, corresponding with a warm November–January period preceding sporulation with the average daily temperature of 4.1 °C (1.8 °C above the long-term average for 1984–2023, SHMÚ). The end of MSS oscillates significantly between the analysed years, from 13 August (2019) to 15 October (2018), as well as the peak date (from the beginning of April 2019 to the end of July 2023). The length of the MSS also showed significant seasonal differences, from 125 days (2019) to 196 days (2018). The SSIn, which, together with the peak value, indicates the intensity of the spore season, ranged between 114 spore*day/m^3^ (2020) and 332 spore*day/m^3^ (2023). The daily peaks ranged from 4 spores/m^3^ recorded in 2018 to 12 spores/m^3^ in 2023 (Table 2).

**Table 2:** Characteristics of the moss Main Spore Season in Bratislava

Characteristics	2018	2019	2020	2021	2022	2023
Spore season start	3 April	11 April	5 April	9 April	5 April	31 March
Spore season end	15 October	13 August	30 August	11 September	7 September	13 September
Season length (days)	196	125	148	156	156	167
SSIn (spore*day/m^3^)	139	123	114	144	231	332
Peak value (spores/m^3^)	4	7	5	11	11	12
Peak day	27 April	11 April	16 June	29 May	5 April	31 July

**Fig. 5:**
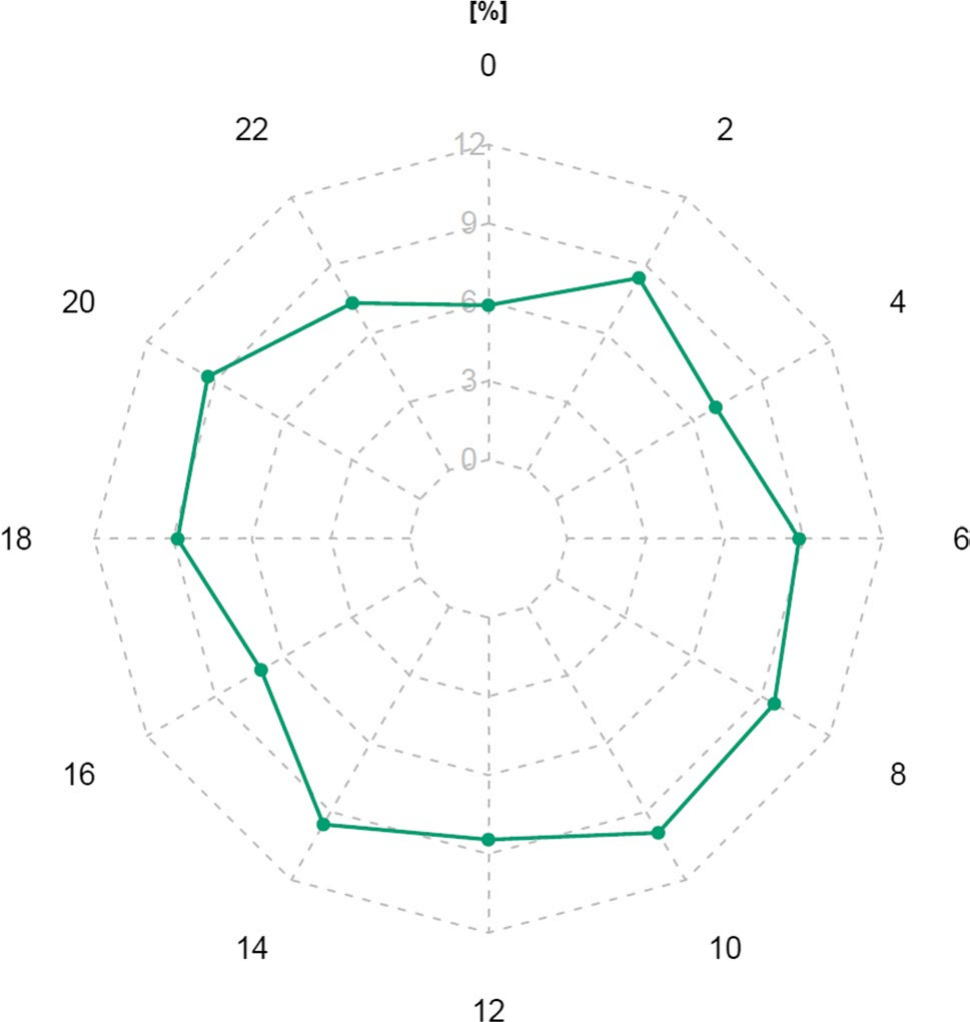
Intradiurnal variation in the moss spore concentration in Bratislava (2018–2023), expressed as percentages

Based on the analysis of intradiurnal variation in spore concentration over the studied years and sites, we found no significant pattern during individual hours of the day (Fig. 5).

**Fig. 6:**
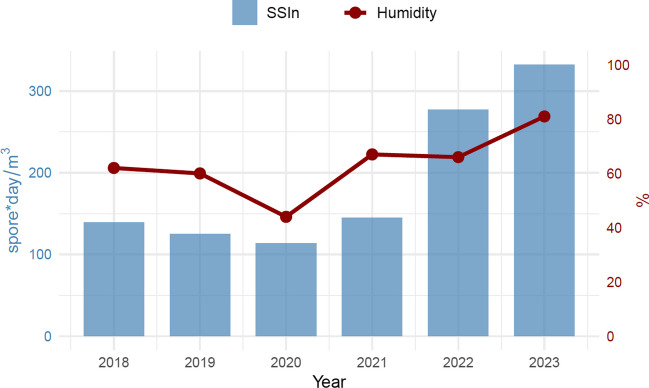
Seasonal Spore Integral (SSIn) and mean daily values of relative humidity in April in Bratislava

The concentration of moss spores in the atmosphere can be influenced by meteorological conditions. Analysis of the studied years showed significant differences in relative humidity in April, influencing the value of SSIn in the given year. The highest SSIn was recorded in 2023, when the mean relative humidity in April reached 81% (27% above the long-term average 1983–2023, SHMÚ), while the 44% relative humidity in April 2020 (31% below the long-term average) was followed by the lowest SSIn (Fig. 6). The release of spores from sporangia and their dispersal is aided by warm weather, which is visible in the difference between July temperatures in 2020 (average daily temperature 21.1 °C, lowest SSIn) and 2023 with highest SSIn and average daily temperature 23 °C (1.3 °C above the long-term average 2000–2023, SHMÚ).

Table 3 shows the results of the Poisson regression analysis of the impacts of environmental variables on airborne moss spore levels. From the analyzed meteorological factors, temperature and wind speed had a positive influence on spore concentrations, while the association with precipitation was negative. Regarding air pollutants, CO concentration was the only significant parameter, influencing the spore concentration negatively.

**Table 3:** Significant environmental variables in Poisson regression model for airborne moss spore concentration in Bratislava over the Main Spore Sason, years 2018−2023

Variables	*β* Coeff.	Std. error	*p*-value
Intercept	−0.9708	0.2976	0.0011
Beta1	0.3788	0.0568	<0.0001
Beta2	0.1437	0.0571	0.0118
Temperature	0.0382	0.0061	<0.0001
Precipitation	−0.008	0.0041	0.0526
Wind speed	0.1242	0.0539	0.0213
CO	−0.0015	0.0006	0.0073
Year2019-2018	0.2147	0.1241	0.0836
Year2020-2018	−0.0232	0.1367	0.8653
Year2021-2018	0.0599	0.1345	0.656
Year2022-2018	0.3789	0.131	0.0038
Year2023-2018	0.6149	0.1255	<0.0001

## Discussion

The spores of mosses, as highly efficient wind-dispersed organisms (see Patiño & Vanderpoorten 2019 for review), can be found in the atmosphere together with pollen grains and fungal spores (Fröhlich-Nowoisky et al. 2016). Since moss spores are not considered allergenic, have relatively low representation in the atmospheric samples (as confirmed by our study) and are difficult to determine based on morphological characteristics, almost no attention was given to them by aerobiologists except for a recent study with regards to evaluating their ability to cope with the impact of the climatic change by higher meiospore production and spreading to more suitable areas (Zanatta et al. 2020).

The airborne moss spores quantity depends on their size, sporangia placement above ground, the size and distribution of source populations and their proximity to the sampling equipment and meteorological conditions. During the six years, airborne moss spores were found frequently but in quite low concentration in the study area. The ASIn ranged from 124 to 367 spore*day/m^3^, with the highest quantity of the size category 13–18 µm. Aerobiological particles of such a small size usually have good aerodynamic properties, which can be aided by the surface perine layer (Glime 2017), but their real concentration does not correspond with the expectations connected with high spore production (Sundberg & Rydin 1998). While pollen grains and fungal spores are dominant in the atmosphere of the study area, the concentration of moss spores is comparable to fern spores (Ščevková et al. 2022, 2023). One of the reasons could be the habitat preference of mosses in the studied area, with their highest occurrence in closed forest canopies (Janovicová et al. 2003; Jančovičová et al. 2022), where vegetation acts as a barrier influencing air circulation. In these conditions, spores usually sediment within a few meters from the source (Lönnell et al. 2015) or adhere to the surrounding plants, which prevents them from getting into the air stream and being captured by the aerobiological samplers, similarly to the results of other researchers (Mota de Oliveira et al. 2022). The argument for the low ability of moss spores to escape closed canopies is further supported by the airflow in Bratislava with prevailing northwestern winds. Despite a general preference of mosses for growth on the northern side of trees (Trynoski & Glime 1982) and the wind blowing from the Carpathian forests (the biggest local source of moss spores) into the city, we still find their concentrations trapped in the sampler quite low.

In Central Europe, many mosses sporulate during the vegetation season between April and September, resulting in the highest airborne spore concentration (Stark 2002; Proctor et al. 2007). The highest spore levels in the study area were recorded in July, which supports the assumption that most of these airborne spores belong to forest species which mostly sporulate during summer due to sufficient shade and moisture during the whole vegetation period. In mosses that grow in less shady areas, the stress caused by drier and warmer conditions can lead to sporulation in the colder part of the year to prevent loss of water through sporophytes (Duckett et al. 2009).

No intradiurnal pattern was observed in the aerobiological data, which can be explained by the heat island effect in an urban environment. The near-surface layer of the troposphere remains dynamic even during the evening and nighttime hours, thus preventing spore sedimentation in the evening (Jones & Harrison 2004).

Regarding the timing and intensity of the MSS, we noted that its start depended mainly on the temperatures in November–January preceding sporulation. Warm temperatures during these months probably initiate earlier growth of sporophytes, similar to the flowering woody plants in spring influencing the onset of their pollen seasons (Schramm et al. 2021). The intensity of MSS, represented by SSIn, was instead influenced by high humidity in April, stimulating the formation of spores in sporangia (Proctor et al. 2007; Glime & Bisang 2017) and high temperatures in July, aiding the dispersal of spores in the air (Proctor et al. 2007; Sundberg 2010).

The results of the statistical analysis show a dependence of airborne moss spore levels on a combination of several environmental parameters. The positive impact of temperature and wind speed and the negative impact of precipitation are similar to their influence on other airborne bioparticles, like pollen grains and fungal spores (Grinn-Gofroń & Bosiacka 2015; Ianovici 2016; Rosas et al. 2020; Schramm et al. 2021; Rahman et al. 2021; Ajikah et al. 2023). The release of moss spores in most temperate climate species is controlled by xerochastic movements of the peristome (Zanatta et al. 2018), thus releasing more spores in warm and dry conditions. The detachment of spores from the boundary layer is influenced by wind speed and air turbulence, initiating sporophyte vibration (Johansson et al. 2014), with the turbulence being also caused by higher temperatures. After their release, the airborne spore levels in the atmosphere can be decreased by the “wash-out” effects of precipitation (Pérez et al. 2009).

CO was found to act as a potent antioxidant in plants, protecting them against oxidative damage caused by biotic and abiotic stressors (Sa et al. 2007). However, we found a negative association of CO with airborne moss spore concentration. The greatest source of this indirect greenhouse gas is incomplete combustion of organic materials, therefore its concentration is highest in colder months of the year during the heating season. The airborne moss spores levels are lowest during this period, so its association with CO is probably a correlation, not causation.

## Conclusions

We have conducted a pilot study of the presence of moss spores in the samples from the aerobiological trap in Bratislava (2018–2023). Our results show a relatively low representation of this type of bioparticles, despite their characteristics suitable for aerial transport, possibly explained by their preference for closed forest habitats from which they rarely enter free stream air transport.

There are several limitations to this type of studies, since the morphological characteristics of Bryophyte spores do not enable accurate identification of genera and their low abundance in the air is an obstacle to metagenomic studies. Without proper identification, it is not possible to determine the possible differences in the responses of individual taxa to environmental factors. Cultivation of spores would be needed for correct identification, but this is not possible from aerobiological samples without some additional spore capture. Since the aerobiological trap placement can have a great role in the ratio of captured local spores to spores originating in long-distance transport, further studies with different sampler placement (including close to the ground in forested areas) are needed to learn more about the aerobiological pathway of moss spores.

It is important to study the presence of Bryophyte spores in the air despite their lack of allergenic molecules (as known so far) since long-distance transport of moss spores is important for colonising new habitats in the light of the recent climatic change and anthropogenic activities impacting them with gradual loss of their preferred habitats.

## Supplementary Information

Below is the link to the electronic supplementary material.

Supplementary file1 (DOCX 24 KB)

## Data Availability

All data generated or analysed during this study are included in this published article and its supplementary information file.

## Abbreviations

MSS: Main Spore Season
SHMÚ: Slovak Hydrometeorological Institute
ASIn: Annual Spore Integral
SSIn: Seasonal Spore Integral

## Footnotes

1 Note that using previous observations in the model created a slight discrepancy at the turn of the year (for example, the first two data points in 2019 were also modelled by the last observations in 2018, even though these data points are more than two days apart) — as far as we know, the software cannot deal with this situation. However, this should have minimal to no effect on the resulting estimates, as this only affects ten data points (less than 1% of the total), and moreover spore concentrations are close to zero at the end of the observation period, so their effect on the response variable is minimal at these critical points.

## References

[CR1] Ajikah LB, Roffe SJ, Neumann FH, Bamford MK, Esterhuizen N, Berman D, Peter J (2023) Meteorological influences on airborne pollen and spores in Johannesburg (Gauteng), South Africa. Aerobiologia 39:363–388. 10.1007/s10453-023-09799-2

[CR2] Barbé M, Fenton NJ, Bergeron Y (2016) So close and yet so far away: long distance dispersal events govern bryophyte metacommunity re-assembly. J Ecol 104:1707–1719. 10.1111/1365-2745.12637

[CR3] Belčáková I, Slámová M, Demovičová Z (2022) Importance of urban green areas in the context of current and future global changes: lessons learned from a case study in Bratislava (Slovakia). Sustainability 14:14740. 10.3390/su142214740

[CR4] Cerff M, Posten C (2012) Enhancing the growth of *Physcomitrella patens* by combination of monochromatic red and blue light—a kinetic study. Biotechnol J 7:527–536. 10.1002/biot.201100044 21751390 10.1002/biot.201100044

[CR5] Duckett JG, Pressel S, Renzaglia KS (2009) Exploding a myth; the capsule dehiscence mechanism and the function of pseudostomata in *Sphagnum*. New Phytol 183:1053–1063. 10.1111/j.1469-8137.2009.02905.x 19552695 10.1111/j.1469-8137.2009.02905.x

[CR6] Erkara IP, Birgi F, Koyuncu O (2018) Spore morphology, taxonomical and ecological importance of Bryophyta from Turkey. Commun. Fac Sci Univ Ank Series C 27:215–223. 10.1501/commuc_0000000217

[CR7] Essien BCh (2019) The environmental, ecological and medical impact of airborne pollen grains, spores of fungi and Pteridophyte and other atmospheric palynomorphs in Akoko Division, Ondo State, Nigeria. Journal of Biotechnology and Biochemistry 5:08–17. 10.9790/264X-0505010817

[CR8] Frahm JP (2001) Biologie der Moose. Springer, Berlin (Verlag) (**978-3-662-57606-9**)

[CR9] Frahm JP (2009) Diversity, dispersal and biogeography of bryophytes (mosses). In: Foissner W, Hawksworth DL (eds) Protist diversity and geographical distribution. Springer, pp 43–50

[CR10] Fröhlich-Nowoisky J, Kampf ChJ, Weber B, Huffman JA, Pöhlker Ch, Andreae MO, Lang-Yona N, Burrows SM, Gunthe SS, Elbert W, Su H, Hoor P, Thines E, Hoffmann T, Després VR, Pöschl U (2016) Bioaerosols in the Earth system: climate, health, and ecosystem interactions. Atmos Res 182:346–376. 10.1016/j.atmosres.2016.07.018

[CR11] Galán C, Cariñanos P, Alcázar P, Domínguez-Vilches E (2007) Spanish aerobiology network (REA): management and quality manual. Servicio de publicaciones de la Universidad de Córdoba, Córdoba

[CR12] Goffinet B, Buck WR (2004) Systematics of the Bryophyta (mosses): from molecules to a revised classification. Molecular Systematics of Bryophytes 98:205–239

[CR13] Grinn-Gofroń A, Bosiacka B (2015) Effects of meteorological factors on the composition of selected fungal spores in the air. Aerobiologia 31:63–72. 10.1007/s10453-014-9347-1 25750477 10.1007/s10453-014-9347-1 PMC4342788

[CR14] Hodgetts NG, Söderström L, Blockeel TL, Caspari S, Ignatov MS, Konstantinova NA et al (2020) An annotated checklist of bryophytes of Europe, Macaronesia and Cyprus. J Bryol 42:1–116. 10.1080/03736687.2019.1694329

[CR15] Hutsemekers V, Dopagne C, Vanderpoorten A (2008) How far and how fast do bryophytes travel at the landscape scale? Divers Distrib 14:483–492. 10.1111/j.1472-4642.2007.00454.x

[CR16] Ianovici N (2016) Atmospheric concentrations of selected allergenic fungal spores in relation to some meteorological factors, in Timişoara (Romania). Aerobiologia 32:139–156. 10.1007/s10453-016-9427-5

[CR17] Jančovičová S, Kubalová S, Mišíková K (2022) Let’s go to the field. Botanical excursion 5. Acta Botanica Universitatis Comenianae 59:19–37

[CR18] Jones AM, Harrison RM (2004) The effects of meteorological factors on atmospheric bioaerosol concentrations: a review. Sci Total Environ 326:151–180. 10.1016/j.scitotenv.2003.11.021 15142773 10.1016/j.scitotenv.2003.11.021

[CR19] Khattab A, Levetin E (2008) Effect of sampling height on the concentration of airborne fungal spores. Ann Allergy Asthma Immunol 101:529–534. 10.1016/S1081-1206(10)60293-1 19055208 10.1016/S1081-1206(10)60293-1

[CR20] Liboschik T, Fokianos K, Fried R (2017) tscount: an R package for analysis of count time series following generalized linear models. J Stat Softw 82:1–51. 10.18637/jss.v082.i05

[CR21] Löbel S, Snäll T, Rydin H (2009) Mating system, reproduction mode and diaspore size affect metacommunity diversity. J Ecol 97:176–185. 10.1111/j.1365-2745.2008.01459.x

[CR22] Lönnell N, Norros V, Sundberg S, Rannik Ü, Johansson V, Ovaskainen O, Hylander K (2015) Testing a mechanistic dispersal model against a dispersal experiment with a wind-dispersed moss. Oikos 124:1232–1240. 10.1111/oik.01886

[CR23] Marshall WA, Convey P (1997) Dispersal of moss propagules on Signy Island, maritime Antarctic. Polar Biol 18:376–383. 10.1007/s003000050203

[CR24] Medina NG, Estébanez B (2014) Does spore ultrastructure mirror different dispersal strategies in mosses? A study of seven Iberian *Orthotrichum* species. PLoS One 9:e112867. 10.1371/journal.pone.0112867 25412450 10.1371/journal.pone.0112867 PMC4239030

[CR25] Miller NG, McDaniel SF (2004) Bryophyte dispersal inferred from colonization of an introduced substratum on Whiteface Mountain, New York. Am J Bot 91:1173–1182. 10.3732/ajb.91.8.1173 21653473 10.3732/ajb.91.8.1173

[CR26] Mota de Oliveira S, Duijm E, Stech M, Ruijgrok J, Polling M, Barbosa CGG, Cerqueira GR, Nascimento AHM, Godoi RHM, Taylor PE, Wolff S, Weber B, Kesselmeier J (2022) Life is in the air: an expedition into the Amazonian atmosphere. Front Ecol Evol 10:789791. 10.3389/fevo.2022.789791

[CR27] Nilsson S, Persson S (1981) Tree pollen spectra in the Stockholm region (Sweden), 1973–1980. Grana 20:179–182. 10.1080/00173138109427661

[CR28] Oksanen E, Kontunen-Soppela S (2021) Plants have different strategies to defend against air pollutants. Curr Opin Environ Sci Health 19:100222. 10.1016/j.coesh.2020.10.010

[CR29] Patiño J, Vanderpoorten A (2019) Bryophyte biogeography. Crit Rev. Plant Sci 37:175–209. 10.1080/07352689.2018.1482444

[CR30] Pérez CF, Gassmann MI, Covi MJA (2009) An evaluation of the airborne pollen–precipitation relationship with the superposed epoch method. Aerobiologia 25:313–320. 10.1007/s10453-009-9135-5

[CR31] Polevova SV, Moiseenko AV, Kolesnikova MA, Ashikhmina DA, Ignatov MS (2019) An attempt to create air sacs in spores? On the unusual spore structure in moss *Encalypta longicollis*. Arctoa 28:171–178. 10.15298/arctoa.28.15

[CR32] Proctor MCF, Oliver MJ, Wood AJ, Alpert P, Stark LR, Cleavitt NL, Mishler BD (2007) Desiccation-tolerance in Bryophytes: a review. The Bryologist 110:595–621. 10.1639/0007-2745(2007)110[595:DIBAR]2.0.CO;2

[CR33] Qian H, Dai Z, Wang J (2024) Strong evidence for latitudinal diversity gradient in mosses across the world. Plant Divers 46:537–541. 10.1016/j.pld.2024.05.004 39280978 10.1016/j.pld.2024.05.004 PMC11390623

[CR34] Rahman A, Khan MHR, Luo Ch, Yang Z, Ke J, Jiang W (2021) Variations in airborne pollen and spores in urban Guangzhou and their relationships with meteorological variables. Heliyon 7:e08379. 10.1016/j.heliyon.2021.e08379 34825088 10.1016/j.heliyon.2021.e08379 PMC8605060

[CR35] Rosas CIO, Calderón-Ezquerro MC, Gutiérrez-Ruacho OG (2020) Fungal spores and pollen are correlated with meteorological variables: effects in human health at Hermosillo, Sonora, Mexico. Int J Environ Health Res 30:677–695. 10.1080/09603123.2019.1625031 31161773 10.1080/09603123.2019.1625031

[CR36] Sa ZS, Huang LQ, Wu GL, Ding JP, Chen XY, Yu T, Shi C, Shen WB (2007) Carbon monoxide: a novel antioxidant against oxidative stress in wheat seedling leaves. J Integr Plant Biol 49:638–645. 10.1111/j.1744-7909.2007.00461.x

[CR37] Ščevková J, Vašková Z, Dušička J, Hrabovský M (2022) Fern spores: neglected airborne bioparticles threatening human health in urban environments. Urban Ecosyst 25:1825–1838. 10.1007/s11252-022-01263-2

[CR38] Ščevková J, Vašková Z, Dušička J, Žilka M, Zvaríková M (2023) Co-occurrence of airborne biological and anthropogenic pollutants in the central European urban ecosystem. Environ Sci Pollut Res 30:26523–26534. 10.1007/s11356-022-24048-8 10.1007/s11356-022-24048-8 PMC9651122 36367655

[CR39] Schramm PJ, Brown CL, Saha S, Conlon KC, Manangan AP, Bell JE, Hess JJ (2021) A systematic review of the effects of temperature and precipitation on pollen concentrations and season timing, and implications for human health. Int J Biometeorol 65:1615–1628. 10.1007/s00484-021-02128-7 33877430 10.1007/s00484-021-02128-7 PMC9016682

[CR40] Snäll T, Ehrlén J, Rydin H (2005) Colonization-extinction dynamics of anepiphyte metapopulation in a dynamic landscape. Ecology 86:106–115. 10.1890/04-0531

[CR41] Spieksma FThM, van Noort P, Nikkels H (2000) Influence of nearby stands of *Artemisia* on street-level versus roof-top-level ratioʼs of airborne pollen quantities. Aerobiologia 16:21–24. 10.1023/A:1007618017071

[CR42] Stark LR (2002) Phenology and its repercussions on the reproductive ecology of mosses. Bryologist 105:204–218. 10.1639/0007-2745(2002)105[0204:PAIROT]2.0CO;2

[CR43] Sundberg S (2010) Size matters for violent discharge height and settling speed of *Sphagnum* spores: important attributes for dispersal potential. Ann Bot 105:291–300. 10.1093/aob/mcp288 20123930 10.1093/aob/mcp288 PMC2814761

[CR44] Sundberg S (2013) Spore rain in relation to regional sources and beyond. Ecography 36:364–373. 10.1111/j.1600-0587.2012.07664.x

[CR45] Sundberg S, Rydin H (1998) Spore number in *Sphagnum* and its dependence on spore and capsule size. J Bryol 20:1–16. 10.1179/jbr.1998.20.1.1

[CR46] Trynoski SE, Glime JM (1982) Direction and height of Bryophytes on four species of northern trees. The Bryologist 85:281–300 (http://www.jstor.org/stable/3243047)

[CR47] Varela Z, Boquete MT, Fernández JA, Martínez-Abaigar J, Núñez-Olivera E, Aboal JR (2023) Mythbusters: unravelling the pollutant uptake processes in mosses for air quality biomonitoring. Ecol Indic 148:110095. 10.1016/j.ecolind.2023.110095

[CR48] Wiklund K, Rydin H (2004) Ecophysiological constraints on spore establishment in bryophytes. Funct Ecol 18:907–913. 10.1111/j.0269-8463.2004.00906.x

[CR49] Zanatta F, Vanderpoorten A, Hedenäs L, Johansson V, Patiño J, Lönnell N, Hylander K (2018) Under which humidity conditions are moss spores released? A comparison between species with perfect and specialized peristomes. Ecol Evol 8:11484–11491. 10.1002/ece3.4579 30598750 10.1002/ece3.4579 PMC6303758

[CR50] Zanatta F, Engler R, Collart F, Broennimann O, Mateo RG, Papp B, Muñoz J, Baurain D, Guisan A, Vanderpoorten A (2020) Bryophytes are predicted to lag behind future climate change despite their high dispersal capacities. Nat Commun 11:5601. 10.1038/s41467-020-19410-8 33154374 10.1038/s41467-020-19410-8 PMC7645420

[CR51] Ziska LH (2021) Climate, carbon dioxide, and plant-based aero-allergens: a deeper botanical perspective. Front Allergy 20:714724. 10.3389/falgy.2021.714724 10.3389/falgy.2021.714724 PMC8974748 35386997

[CR52] Boros Á, Járai-Komlódi M, Tóth Z, & Nilsson S (1993) An atlas of recent European Bryophyte spores. Scientia Publishing, 321 p.

[CR53] Glime JM, & Bisang I (2017) Sexuality: sex ratio and sex expression. Chapt. 3-2. In: Glime J M. Bryophyte Ecology. Volume 1. Physiological Ecology. Accessed March 11, 2024 from http://www.bryoecol.mtu.edu.

[CR54] Glime JM (2017) Adaptive strategies: vegetative vs sexual diaspores. Chapt. 4-7. In: Glime JM. Bryophyte Ecology. Volume 1. Physiological Ecology. Accessed February 29, 2024 from http://www.bryoecol.mtu.edu.

[CR55] Halbritter H, Sam S, & Auer W (2020)&nbsp;Artemisia vulgaris. In: PalDat - A palynological database. https://www.paldat.org/pub/Artemisia_vulgaris/304632; accessed 2024-07-22.

[CR56] Janovicová K, Kubinská A, & Javorčíková D (2003) Liverworts (Hepatophyta), rosettes (Anthocerotophyta) and mosses (Bryophyta) in the territory of Bratislava./Pečeňovky (Hepatophyta), rožteky (Anthocerotophyta) a machy (Bryophyta) na území Bratislavy. Botanický ústav SAV, Bratislava (in Slovak).

[CR57] Johansson V, Lönnell N, Sundberg S, & Hylander K (2014) Release thresholds for moss spores: the importance of turbulence and sporophyte length. J Ecol 102: 721–729. https://www.jstor.org/stable/24541426.

[CR58] Lacey ME, & West JS (2006) The air spora. The manual for catching and identifying airborne biological particles. Dordrecht, the Netherlands: Springer-Verlag Gmbh. 10.1111/j.1365-3059.2007.01610.x.

[CR59] Martínez-Abaigar J, & Núñez-Olivera E (2021) Chapter 11 - Novel biotechnological substances from bryophytes. In: Sinha RP, Häder DP (Eds.) Natural Bioactive Compounds. Academic Press: 233–248. 10.1016/B978-0-12-820655-3.00011-2.

