# Supplementary material for: Moss spores: overlooked airborne bioparticles in an urban environment
Source: Environ Sci Pollut Res Int. 2024 Sep 21;31(47):58010–20. doi: 10.1007/s11356-024-35028-5 (PMC11467093; doi:10.1007/s11356-024-35028-5)
Supplement: Supplementary file 1 — Supplementary file1 (DOCX 24 KB) [file 11356_2024_35028_MOESM1_ESM.docx]

**Moss spores: overlooked airborne bioparticles in an urban environment**

Jana Ščevková, Mária Tropeková, Jozef Dušička, Natália Štefániková, Matúš Žilka, Eva Zahradníková, Jozef Kováč, Katarína Mišíková

**SUPPLEMENTARY INFORMATION:**

**Table S1** Spore morphological characteristics of abundantly represented mosses in Bratislava

| Species^a^ | Spore size (µm) | | Exine  thickness  (µm) | Exine surface^b^ | Ecological group | Sporulating period  (start – end) |
| --- | --- | --- | --- | --- | --- | --- |
|  | Mean value | Range |  |  |  |  |
| *Amblystegium serpens* | 13.1 | 15.6–10.4 | < 1 | structured | ubiquitous | III. – VIII. |
| *Atrichum undulatum* | 20.3 | 25.1–17.4 | ~ 1 | structured | epigeic | XI. – IV. |
| *Barbula unguiculata* | 18.8 | 21.9–17.0 | ~ 1.5 | structured | epigeic | XI. – IV. |
| *Brachytheciastrum velutinum* | 11.5 | 14.5–8.8 | ~ 0.5 | structured | ubiquitous | I. – IV. |
| *Brachythecium rutabulum* | 14.8 | 16.6–12.4 | ~ 0.8 | delicately structured | ubiquitous | XI. – V. |
| *Bryum argenteum* | 10.5 | 8.0–14.0 | < 0.5 | smooth | epigeic/epilithic | XI. - V. |
| *Ceratodon purpureus* | 13.1 × 11.9 | 13.9–10.8 | ~ 0.8 | structured | epigeic | V. – VII. |
| *Funaria hygrometrica* | 15.7 | 18.8–12.5 | ~ 1 | structured | epigeic | I. – XII. |
| *Grimmia pulvinata* | 11.1 | 15.7–9.8 | ~ 0.8 | delicately structured | epilithic | III. – IX. |
| *Hypnum cupressiforme* | 15.4 | 12.0–20.0 | ~ 0.8 | delicately structured | ubiquitous | XI. – III. |
| *Leskea polycarpa* | 14.4 | 18.7–9.8 | < 0.5 | delicately structured | epiphyte | V. – VIII. |
| *Lewinskya affinis* | 19.6 | 16.0–24.0 | ~ 2 | structured | epiphyte | IV. – IX. |
| *Orthotrichum* *diaphanum* | 15.7 | 14.0–18.0 | ~ 1.5 | structured | epiphyte | I. – XII. |
| *Polytrichum formosum* | 9.3 | 11.9–7.7 | ~ 0.2 | smooth | epigeic | V. – VIII. |
| *Schistidium* *apocarpum* | 12.6 × 10.3 | 15.7–9.7 | ~ 0.6 | delicately structured | epilithic | II. – III. |
| *Tortula muralis* | 10.6 | 12.4–9.3 | ~ 1 | structured | epilithic | V. – X. |

^a^The nomenclature of mosses follows Hodgetts et al. (2020)

^b^Surface structures are usually verrucate or pilum-like
